# Deorphanisation and functional characterisation of OATP5A1 as transport protein for amino acids and vitamins

**DOI:** 10.1186/s11658-026-00943-7

**Published:** 2026-06-02

**Authors:** Elena Kohlmann, Nikola K. Schmid, Arne Gessner, Martin F. Fromm, Jörg König

**Affiliations:** 1 Institute of Experimental and Clinical Pharmacology and Toxicology, Friedrich-Alexander-Universität Erlangen-Nürnberg, Erlangen, Germany; 2 FAU NeW–Research Center New Bioactive Compounds, Friedrich-Alexander-Universität Erlangen-Nürnberg, Erlangen, Germany

**Keywords:** Orphan transporter, Solute carrier, OATP, OATP5A1, Transport, Amino acids, Thiamine, Tyrosine, Glutamine, Glycine

## Abstract

**Background:**

Transport proteins are important for the uptake, distribution and elimination of endogenous substances and drugs, and therefore essential for, e.g., cellular metabolism or drug effects. While the export of substrates out of cells is mediated by ATP-binding cassette (ABC) transporters, SLC (solute carrier) transporters are mainly responsible for the uptake into cells. In contrast to most well-characterised ABC transporters, many SLC transporters have been studied insufficiently. Such transporters are called orphan transporters. Despite the fact that the *SLC21*/*SLCO* family contains several important transporters for widely prescribed drugs, one of its family members, OATP5A1 (*SLCO5A1*), is such an orphan transporter. OATP5A1 is ubiquitously expressed throughout the body, including expression in the brain, heart, intestine and various cancerous tissues. However, no substrates have been characterised for this transporter to date.

**Methods:**

Using stably-transfected HEK293 cells overexpressing human OATP5A1 (HEK-OATP5A1) and the respective control cells (HEK-VC), we investigated known substrates of other OATP family members as potential OATP5A1 substrates. Furthermore, an untargeted metabolomics analysis of both cell lines was performed after incubation with human plasma. Candidate substances were further characterised as substrates of OATP5A1.

**Results:**

After characterisation of the stably-transfected HEK-OATP5A1 cells, uptake assays and untargeted metabolomics analysis identified the hormone conjugate estrone-3-sulfate, the amino acids glutamine, glycine and tyrosine, the vitamins pantothenic acid (vitamin B_5_) and thiamine (vitamin B_1_) and the nucleotide thymine as potential OATP5A1 substrates. While estrone-3-sulfate, tyrosine and thiamine were further characterised as uptake substrates, glutamine and glycine were exported by OATP5A1. Moreover, pantothenic acid and thymine inhibited OATP5A1-mediated tyrosine uptake. For estrone-3-sulfate, tyrosine and thiamine, kinetic transport parameters (K_m_ values) of 102.2 µM, 169.9 µM and 15.6 µM were calculated, respectively.

**Conclusions:**

In the present study, OATP5A1 was deorphanised by characterising amino acids and vitamins as substrates of this transport protein. Estrone-3-sulfate, tyrosine and thiamine were taken up by OATP5A1. Moreover, OATP5A1 mediated the efflux of the amino acids glutamine and glycine, which play essential roles in brain function.

**Graphical Abstract:**

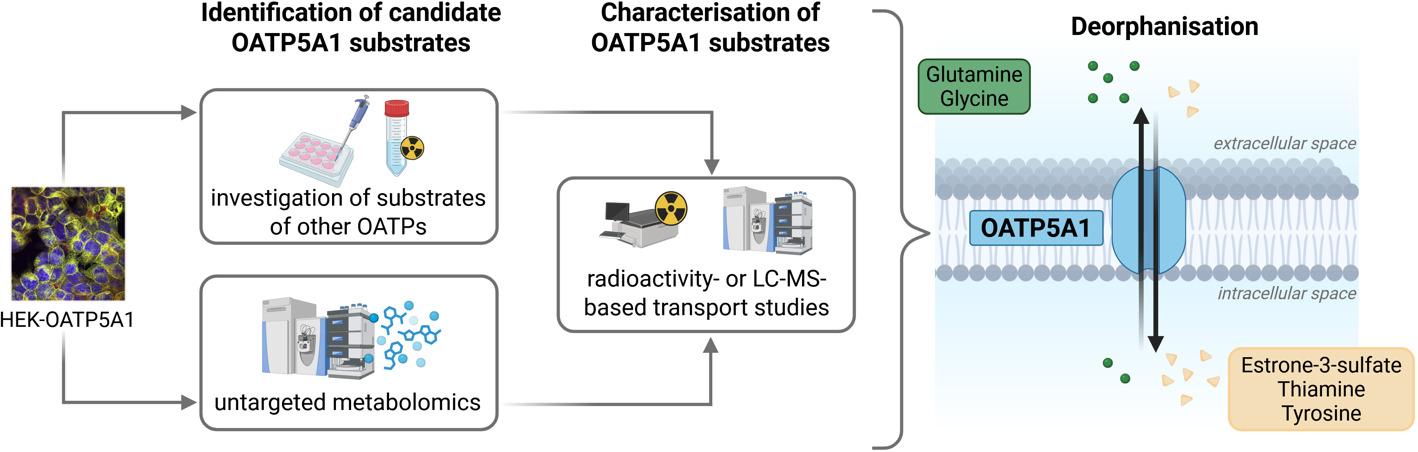

**Supplementary Information:**

The online version contains supplementary material available at 10.1186/s11658-026-00943-7.

## Introduction

Transport proteins play an important role in cellular metabolism by mediating the uptake of substances into or the export of substances out of cells and are classified as either ABC (ATP-binding cassette) efflux transporters or as SLC (solute carrier) transporters that mainly act as uptake transporters [1]. A total of 48 ABC transporters have been identified in humans, and their physiological role as well as their involvement in transporting drugs and drug metabolites is well characterised [2]. The SLC transporter superfamily consists of more than 400 transport proteins grouped into 70 different families [3]. As shown for ABC transporters, SLC transporters also mediate the transport of endogenous substances and metabolites across membranes and cellular compartments. However, in contrast to ABC transporters, most SLC transporters act as uptake transporters. In addition, several SLC transporters are drug targets or characterised as drug transporters [1, 4, 5]. Despite their importance for the transport of endogenous substances and xenobiotics, the knowledge on the substrate spectrum of approximately 30% of SLC transporters is limited. Consequently, these transporters are designated as orphan transporters [3]. Furthermore, systematic in silico studies of the human genome revealed that more than 120 human SLC or SLC-like transporters are currently unidentified and uncharacterised [6].

SLC transporter families of particular importance for drug disposition and effects are the *SLC21*/*SLCO* family and the *SLC22* family. The *SLC22* family consists of 28 human members, including transporters for organic anions (OATs) and organic cations (OCTs), and contains 10 orphan transport proteins [7]. The *SLC21*/*SLCO* family of organic anion transporting polypeptides (OATPs) includes 11 human family members, grouped into the subfamilies OATP1 to OATP6. OATPs exhibit a broad and partially overlapping substrate spectrum, mediating the uptake of drugs, amino acids and their derivatives, hormones and hormone metabolites and bile acids [8–10]. The family members OATP1B1 (*SLCO1B1*) and OATP1B3 (*SLCO1B3*) are both highly expressed in hepatocytes. Besides endogenous compounds, several widely prescribed drugs are substrates of OATP1B1 and OATP1B3, including hydroxymethylglutaryl-coenzyme A reductase inhibitors (statins), antibiotics and antihypertensive drugs. For an overview about the substrate and drug spectrum transported by OATP1B1 and OATP1B3 see recent reviews [4, 8, 11]. Therefore, according to the international guidelines of the Food and Drug Administration (FDA), the European Medicines Agency (EMA) and the Japanese Pharmaceuticals and Medical Devices Agency (PMDA), these transport proteins are among those that should be investigated during drug development [12]. Considering this, it is remarkable that there are transporters belonging to the OATP family that are still orphan transporters without any substance being characterised as substrate for these members.

One of these orphan OATP family members is OATP5A1 (*SLCO5A1*). Similar to other OATP family members, OATP5A1 has 12 predicted transmembrane helices with intracellularly located amino- and carboxyterminals [8, 13]. Based on binding site analysis, OATP5A1 clusters together with OATP4A1, OATP4C1 and OATP6A1 into class 3 OATPs. Furthermore, OATP5A1 exhibits a druggable inner pore characteristic for human OATPs [13].

Despite the fact that expression analysis demonstrated that OATP5A1 is ubiquitously expressed in healthy tissues, including higher expression in the brain, breast, dendritic cells, heart and placenta [14–19], no substrates have been characterised for this transporter thus far. Furthermore, OATP5A1 is overexpressed in various cancerous tissues [14, 18–20] and studies indicate that higher *SLCO5A1* mRNA expression is a prognostic marker for overall survival in ovarian cancer [21] and for poor outcome in renal carcinoma [22] and uveal melanoma [23]. In addition, a single nucleotide polymorphism (SNP) that colocalises with the *SLCO5A1* gene contributes to impulsivity in juvenile myoclonic epilepsy [24]. These data suggest that OATP5A1 plays an important role in several (patho-) physiological processes throughout the body and emphasises the necessity for the further characterisation of this so far orphan transport protein.

Therefore, we characterised stably-transfected HEK-OATP5A1 cells and the respective HEK-VC control cells. Using these cells and standardised uptake assay protocols, substances identified as substrates for other OATP family members were tested as potential OATP5A1 substrates. In addition, an untargeted metabolomics analysis was performed after incubating the cells with human plasma and several amino acids as well as vitamins were discovered and characterised as substrates for OATP5A1.

## Materials and methods

### Chemicals

For radioactive uptake assays, all labelled chemicals were obtained from American Radiolabeled Chemicals, Inc. (St. Louis, MO, USA) unless indicated otherwise. [^3^H]labelled phenylalanin (1 mCi/mL; 60 Ci/mmol) was procured from Hartmann Analytic GmbH (Braunschweig, Germany). [2-^3^H]labelled glycine (1 mCi/mL; 30 Ci/mmol) and [^3^H(G)]labelled pravastatin (1 mCi/mL; 30 Ci/mmol) were from Moravek Biochemicals (Brea, CA, USA). The recovery standards 2-fluorophenylglycine, 4-chlorophenylalanine, the internal standard ^13^C_4_-thiamine hydrochloride and unlabelled benzbromarone, estrone-3-sulfate potassium salt, estradiol-17β-glucuronide, pantothenic acid, rifampicin, thiamine hydrochloride, thymine and DABCO 33-LV were purchased from Sigma-Aldrich Chemie GmbH (Taufkirchen, Germany). All unlabelled amino acids, with the exception of glutamine and glycine, were obtained from Merck KGaA (Darmstadt, Germany). Atorvastatin calcium hydrate and rosuvastatin calcium were from MedChemExpress (Sollentuna, Sweden). Bromosulfophthalein (BSP) was obtained from AppliChem (Darmstadt, Germany) and cyclosporine A from LC Laboratories (Woburn, MA, USA). Glutamine, glycine, sodium chloride and sodium dodecyl sulfate, bovine serum albumin, Triton X 100, Tween 20, Mowiol 4–88 and 5% non-fat milk powder were acquired from Carl Roth GmbH + Co. KG (Karlsruhe, Germany). Pravastatin was obtained from Bio Trend (Köln, Germany) and prostaglandin E_2_ (PGE_2_) from Biomol GmbH (Hamburg, Germany). Liquid chromatography-mass spectrometer (LC–MS) grade acetonitrile, ammonium formate, formic acid, methanol and water were acquired from VWR chemicals (Darmstadt, Germany).

### Cell culture

Unless stated otherwise, all reagents for cell culture were purchased from Life Technologies GmbH (Darmstadt, Germany). Human embryonic kidney 293 (HEK293) cells (American Type Culture Collection ATCC, Manassas, VA, USA) were cultivated at 37 °C and 5% CO_2_ in minimal essential medium (MEM, Gibco by Thermo Fisher Scientific, Waltham, MA, USA) supplemented with 10% heat-inactivated foetal bovine serum, 100 U/mL penicillin, 100 µg/mL streptomycin and 800 µg/mL geneticin. The cells were routinely subcultured at a ratio of 1:3 on two occasions per week after incubation with a 0.05% trypsin-0.02% ethylenediaminetetraacetic acid solution. The cells were examined at regular intervals for mycoplasma contamination using the Venor GeM OneStep Mycoplasma Detection Kit (Minerva Biolabs GmbH, Berlin, Germany*)* and for the stable expression of the *SLCO5A1* mRNA by qRT-PCR.

### Establishment of HEK293 stably overexpressing OATP5A1

HEK293 cells stably overexpressing OATP5A1 have previously been established by our group and were used in other studies [17], but have not been fully characterised thus far. In brief, commercially available total RNA isolated from human heart served as the template for cloning the *SLCO5A1* cDNA encoding human OATP5A1 by a reverse transcription-polymerase chain reaction (RT-PCR) approach. Primers were purchased from Sigma-Aldrich Chemie GmbH (Taufkirchen, Germany; see Table 1 for primer sequences). The *SLCO5A1* cDNA was cloned into the pCR2.1.TOPO vector and subcloned into the expression vector pcDNA3.1(+) (both from Invitrogen by Thermo Fisher Scientific, Waltham, MA, USA) resulting in the plasmid pOATP5A1.31. The sequence of the cloned cDNA was verified and base pair exchanges leading to amino acid exchanges after translation were corrected using the QuikChange Multi Site-Directed Mutagenesis Kit (Agilent Technologies, Santa Clara, CA, USA) resulting in a protein that is 100% identical to the reference protein (NP_112220.2). HEK293 cells were transfected with the pOATP5A1.31 plasmid using the Effectene transfection reagent (QIAGEN, Hilden, Germany) and the cell clone with the highest *SLCO5A1* mRNA expression was identified by quantitative RT-PCR (qRT-PCR). This clone was designated as HEK-OATP5A1. HEK293 cells transfected with the empty vector and selected under the same conditions have already been established [25] and served as control cells (HEK-VC). In addition, both cell lines were analysed for the expression of additional transport proteins by quantifying the mRNA amount of seven human OATP family members and seven transporters known to transport amino acids or vitamins (Additional file 3).

**Table 1:** Primer sequences for molecular cloning and qPCR

	Forward primer	Reverse primer	Fragment length
SLCO5A1.part	5ʹ-CAA CAG GAA TGT GGT GTG CAG-3ʹ	5ʹ-AGC TTC AGG AGG GCG GCT C-3ʹ	403 bp
SLCO5A1.full-length	5ʹ-CTA AGC GCC ATG GAC GAA GG-3ʹ	5ʹ-AGC TTC AGG AGG GCG GCT C-3ʹ	2560 bp
ACTB.part	5ʹ-TGA CGG GGT CAC CCA CAC TGT GCC CAT CTA-3ʹ	5ʹ-CTA GAA GCA TTT GCG GTG GAC GAT GGA GGG-3ʹ	661 bp

Primer used for qPCR-analysis (.part) and molecular cloning (.full-length). bp, base pairs

### Quantitative polymerase chain reaction

qRT-PCR analysis was performed as described before [14]. In summary, mRNA of the transfected cells and the control cells was isolated using the NucleoSpin RNA Plus Mini Kit (Macherey–Nagel, Düren, Germany) according to the manufacturer’s protocol. Subsequently, 1 µg total RNA per sample was reverse transcribed to generate sscDNA by the LunaScript RT SuperMix Kit (New England Biolabs, Ipswitch, MA, USA). The LightCycler 2.0 Instrument and the LightCycler FastStart DNA Master^PLUS^ SYBR Green I reagent (both from Roche Diagnostics GmbH, Mannheim, Germany) were used for the quantification of *SLCO5A1* mRNA. The expression of *SLCO5A1* was calculated with linear regression using a calibration curve of known plasmid dilutions containing the *SLCO5A1* cDNA and related to the expression of the housekeeping gene *ACTB*, which encodes the protein β-actin in the respective cell line. The primers of *ACTB* were obtained from biomers.net (Ulm, Germany; see Table 1 for primer sequence).

### Immunoblot analysis

Immunoblot analysis was performed as described previously [26]. In summary, HEK-OATP5A1 cells and HEK-VC cells were seeded in Cellstar cell culture dishes (Greiner Bio-One GmbH, Frickenhausen, Germany). At 24 h after seeding, the cells were induced with MEM supplemented with 10 mM sodium-butyrate (Merck KGa, Darmstadt, Germany). Following a 24 h period, the cells were detached from the dishes using a cell scraper, centrifuged (10 min, 400 *g*, 4 °C) and resuspended in 0.2% sodium dodecyl sulphate containing mini-complete protease inhibitor cocktail tablets (Roche Diagnostics, Mannheim, Germany). Protein concentrations were measured photometrically with the Pierce BCA Protein Assay Reagent (Thermo Fisher Scientific, Waltham, MA, USA). An amount of 15 µg total protein from each cell line was incubated with Laemmli buffer at 95 °C for 5 min and applied to a 10% sodium dodecyl sulphate (SDS)-polyacrylamide gel. The molecular weight of the protein of interest was evaluated by the PageRuler Plus Prestained Protein Ladder (Thermo Fisher Scientific, Waltham, MA, USA). The proteins were transferred onto a nitrocellulose membrane and blocked with 5% non-fat milk (Carl Roth GmbH + Co. KG, Karlsruhe, Germany). A rabbit polyclonal antiserum SSS, which recognises the C-terminal sequence of OATP5A1 (SSS ADP GLE ESP AAL EPP S, amino acids 830–848), was used as primary antibody for OATP5A1. This antiserum was kindly provided by Prof. Dr. D. Keppler (formerly German Cancer Research Centre, Division of Tumour Biochemistry, Heidelberg). The secondary antibody was an HRP-conjugated goat anti-rabbit IgG antibody (GE Healthcare, Chicago, IL, USA). A monoclonal mouse antibody against β-actin (clone AC-15, Sigma-Aldrich Chemie GmbH, Taufkirchen, Germany) was used as control for equal gel loading. The secondary antibody used for detecting β-actin was an HRP-conjugated goat anti-mouse IgG (Invitrogen by Thermo Fisher Scientific, Waltham, MA, USA). The membrane was incubated overnight at 4 °C with the primary antibody (diluted 1:5000) followed by incubation with the secondary antibody (diluted 1:10,000) for 45 min at room temperature. For the analysis of the β-actin expression, the membrane was stripped with the Restore Western Blot Stripping Buffer (Thermo Scientific, Waltham, MA, USA). The detection was conducted using the Pierce ECL Western Blot Kit (Thermo Scientific, Waltham, MA, USA) and a ChemiDoc XRS detection system (Bio-Rad Laboratories GmbH, Feldkirchen, Germany).

### Immunofluorescence microscopy

The immunofluorescence analysis was conducted as previously described [14, 27]. In detail, the cells were seeded at a density of 2.5 × 10^5^ cells per well onto poly-D-lysine-coated object slides (Thermo Fisher Scientific, Waltham, MA, USA). After 48 h, the cells were washed three times with prewarmed phosphate buffered saline (PBS, Life Technologies GmbH, Darmstadt, Germany) and then fixed with 70% methanol at 4 °C for 10 min. The cells were then again washed three times with PBS and blocked with a 2% aqueous bovine serum albumin (BSA) solution for 45 min. Then, 300 µL of a primary antibody solution, which contained a 1:500 dilution of polyclonal SSS antiserum and a 1:500 dilution of monoclonal alpha-1 sodium–potassium ATPase antibody (Abcam, Cambridge, UK) in 2% aqueous BSA, were added to each well and incubated overnight at 4 °C. The cells were then washed stepwise with PBS-Triton X-100 (0.1%), PBS-Tween 20 (0.05%) and PBS-Triton-X 100 (0.1%) for 5 min, respectively. 4′,6-Diamidin-2-phenylindol (DAPI; 1 mg/mL dissolved in water; AppliChem, Darmstadt, Germany) was used to stain cell nuclei. An Alexa Fluor 568 goat anti-rabbit IgG (A11011) and an Alexa Fluor 647 goat anti-mouse IgG (A32728) (both from Thermo Fisher Scientific, Waltham, MA, USA) were used as secondary antibodies. These antibodies and the DAPI-solution were added to the cells at a dilution of 1:1000 in a 300 µL solution of 2% aqueous BSA. The wells were then washed stepwise with 1 mL of the following reagents: PBS-Tween 20 (0.05%), PBS-Triton X-100 (0.1%) and water. An amount of 10 µL of an antifade mounting medium (6 g glycerol, 2.4 g Mowiol 4–88, 6 mL water, 12 mL Tris buffer pH 8, 2.5% DABCO 33-LV) was used for each slide, on which the cover glass was placed on top. Detection was performed using a Leica Stellaris 8 confocal laser scanning microscope (Leica Microsystems GmbH, Wetzlar, Germany, used at the Optical Imaging Competence Center OICE, Erlangen) with the HC PL APO 63x/1.30 GLYC CORR CS2 objective (Leica Microsystems GmbH, Wetzlar, Germany). Images were further processed using OMERO.web 5.29.0.

### LC–MS untargeted metabolomics

The untargeted metabolomics analysis was performed as described previously [9, 28] with minor modifications. In brief, the cells were seeded and induced 24 h afterwards. At 48 h after seeding, an aliquot of the cells was incubated with prewarmed uptake buffer for 30 min at 37 °C. Subsequently, the uptake buffer was replaced with 300 µL of commercially available pooled human plasma from healthy individuals (BioIVT, West Sussex, UK) for 10 min at 37 °C (treated condition). A second aliquot was processed immediately without any incubation steps (untreated condition).

The cells were then washed three times with an ice-cold 0.9% NaCl solution and lysed using 80% methanol containing the recovery standards DL-4-chlorophenylalanine (0.02 µg/mL) and DL-2-fluorophenylglycine (1.25 µg/mL). After vortexing and centrifugation of the cell lysates (5 min, 4 °C, 24,500 *g*), 300 µL of the supernatant from each sample were used for analysis. In addition, 300 µL homogenised pooled samples were used for quality control. The samples and quality controls were dried under nitrogen at 30 °C and reconstituted in 50 µL of eluent for hydrophilic interaction liquid chromatography (HILIC) analysis or in eluent of reversed phase liquid chromatography (RP) containing internal standards. All samples were measured using an Ultimate 3000 LC-QExactive Focus MS system (Thermo Fisher Scientific, Waltham, MA, USA). Detection was performed in both positive and negative ionisation modes. HILIC and RP chromatography were conducted using either an Acquity UPLC BEH C18 column (1.7 µm, 2.1 × 100 mm) or an Acquity Premier BEH Amide (1.7 µm 2.1 × 100 mm). Both columns were equipped with a 2.1 × 5 mm guard column (Waters, Eschborn, Germany).

### Transport studies

Uptake assays were performed using a standardised protocol [29]. Briefly, cells were seeded with an initial density of 7 × 10^5^ cells per well in poly-D-lysine-coated 12-well cell culture plates. After cultivation for 24 h (37 °C, 5% CO_2_) with MEM, the protein expression was induced using MEM supplemented with 10 mM sodium-butyrate. At 48 h after seeding, transport experiments were performed. Cells were washed with prewarmed uptake buffer (5 mM KCl, 1 mM K_2_HPO_4_, 142 mM NaCl, 1.5 mM CaCl_2_, 1.2 mM MgSO_4_, 5 mM glucose and 12.5 mM HEPES, pH 7.3) and incubated at 37 °C with 300 µL of incubation buffer containing a defined ratio of unlabelled and labelled substrate in uptake buffer. After incubation for a defined time period, the cells were washed three times with ice-cold uptake buffer and lysed with 800 µL 0.2% sodium dodecyl sulfate (SDS) (Carl Roth GmbH + Co. KG, Karlsruhe, Germany).

The radioactivity in each sample was measured by liquid scintillation counting (TriCarb 2800; PerkinElmer Life Sciences GmbH, Rodgau-Jügesheim, Germany) with 500 µL of the sample mixed with 4 mL of Ultima Gold (Revvity Inc., Waltham, MA, USA). The uptake values of each sample were normalised to its protein concentration, which was determined using the bicinchoninic acid assay with the Pierce BCA Protein Assay Reagent from Thermo Fisher Scientific (Waltham, MA, USA).

For the uptake studies using thiamine as substrate, unlabelled thiamine was quantified by LC–MS. Therefore, the cells were induced 24 h after seeding with Dulbecco’s Modified Eagle’s Medium High Glucose without thiamine (United States Biological, Salem, MA, USA) supplemented with 10 mM sodium-butyrate to ensure low intracellular thiamine levels at the onset of the uptake assay. At 48 h after seeding, the uptake assay with unlabelled thiamine was conducted. The cells were then washed three times with precooled 0.9% NaCl solution and then lysed with 500 µL of 80% methanol including the internal standard (0.05 µg/mL ^13^C_4_-labelled thiamine). The samples were then centrifuged (3 min, 24,500 *g*, 4 °C) and 20 µL of the supernatant were mixed with 100 µL of 80% methanol. After centrifuging (3 min, 24,500 *g*, 4 °C), 100 µL of the supernatant were mixed with 400 µL of the eluent (750 mL acetonitrile, 250 mL water, 4 g/L of ammonium formate, pH 3.0). The intracellular concentration of thiamine in each sample was evaluated by LC–MS analysis (Agilent 1100; Agilent Technologies, Waldbronn, Germany and API 4000; Applied Biosystems, Darmstadt, Germany) operating in positive ionisation mode. A volume of 15 µL of the prepared solution was used for injection. The samples were measured using a flow rate of 0.25 mL/min and isocratic mobile phase conditions and a column oven temperature of 30 °C. Chromatographic separation was achieved on an EC 150/2 Nucleoshell HILIC 2.7 µL column in combination with an EC 4/2 Nucleoshell HILIC 2.7 µm guard column (both obtained from Macherey–Nagel, Düren, Germany). A calibration curve was used to quantify the intracellular amounts of thiamine. Therefore, 2 µL of thiamine stock solutions with defined concentrations were mixed with 20 µL of matrix (untreated lysed cells and internal standard in 80% methanol) and 98 µL of 80% methanol. These samples were then centrifuged and diluted with the eluent as described above.

Initial uptake experiments of candidate substrates (estradiol-17β-glucuronide, estrone-3-sulfate, prostaglandin E_2_, atorvastatin, pravastatin, rosuvastatin, arginine, glutamine, glycine, phenylalanine, tryptophan and tyrosine) were performed with an incubation time of 10 min with two concentrations of each substance.

Time-dependent uptake of estrone-3-sulfate, tyrosine and thiamine was assessed after incubation for 1, 2 and 5 min, respectively. For glutamine and glycine, the time-dependent transport was evaluated after incubation for 1, 2, 5, 10 and 20 min.

The determination of the kinetic transport constants (K_m_ values) and maximal transport velocities (V_max_ values) were conducted after 2 min of incubation with substrate concentrations ranging from 1 µM to 250 µM (estrone-3-sulfate and thiamine) or 1 µM to 500 µM (tyrosine). To confirm transporter-mediated uptake of estrone-3-sulfate, tyrosine, glutamine and glycine, the assays were also performed with the same setup and an incubation at 4 °C for 2 min.

The inhibition of tyrosine uptake into HEK-OATP5A1 cells by pantothenic acid and thymine was measured after a 2-min incubation time using 100 µM of tyrosine as substrate and 1 mM of the respective putative inhibitor. In addition, tyrosine uptake was also analysed by adding the prototypic OATP substrates and inhibitors bromosulfophthalein (BSP), benzbromarone, cyclosporine A and rifampicin. All substances were added in the same concentrations of 100 µM and 1 mM, respectively.

For the glutamine efflux assay, the cells were preloaded with 300 µL of a defined ratio of labelled and unlabelled glutamine (2 mM). For the glycine efflux assay, a defined ratio of labelled and unlabelled glycine (2 mM) was used. After 60 min of incubation, one aliquot of cells was washed and lysed to determine the initial intracellular concentrations of the respective amino acid. Subsequently, a second aliquot of cells was incubated with uptake buffer for 2, 5 and 10 min. After each time period, the radioactivity in 500 µL of the supernatant was measured and calculated as percentage of the initial intracellular radioactivity. To verify the transporter-mediated efflux, the assays were additionally carried out using the same setup but with a 2-min incubation at 4 °C.

### Data analysis

The transport experiments were performed with at least six independent biological replicates. Net uptake was calculated by subtracting the uptake values obtained in HEK-VC cells from those into HEK-OATP5A1 cells. The data are expressed as the mean ± standard error of the mean (SEM). A *p*-value of < 0.05 was considered statistically significant. GraphPad Prism 10 (GraphPad Software, San Diego, CA, USA) was used to conduct an unpaired Student’s *t*-test and to determine kinetic transport parameters (K_m_ and V_max_ values) using nonlinear regression with a Michaelis–Menten enzyme kinetics curve fit.

For the untargeted metabolomics assay, five biological replicates of each cell line were used. Data from the metabolomics analysis were further processed using the Compound Discoverer 3.3 Software (Thermo Fisher Scientific, Dreieich, Germany). A compound was considered to have a confirmed structure if it met the criteria for level 1 of identification confidence levels in metabolomics. Therefore, it matches with authentic reference standards in an in-house library in terms of MS-spectra and retention time [30]. Substances showing identification level 1, a log2-fold change > 1 and a *p*-value < 0.05 were further analysed. Log2-fold changes were calculated with the ratio of the quality control-normalised peak area in experiments using HEK-OATP5A1 cells normalised to its protein concentration of control experiments (numerator) and the quality control-normalised peak area in HEK-VC cells normalised to its protein concentration of control experiments (denominator).

Thiamine concentrations were calculated with the area ratios between the peak area of the analyte and the internal standard. Absolute concentrations in the samples were calculated with a calibration curve and normalised to mean protein concentrations of control experiments. To evaluate the uptake of thiamine into cells, the mean intracellular concentrations of untreated cells was subtracted from the intracellular concentration values after incubation with thiamine. For the efflux assays, intracellular radioactivity after preloading was set to 100%, with all subsequent values relating to this initial concentration.

## Results

### Characterisation of stably transfected HEK293 cells overexpressing OATP5A1

**Fig. 1:**
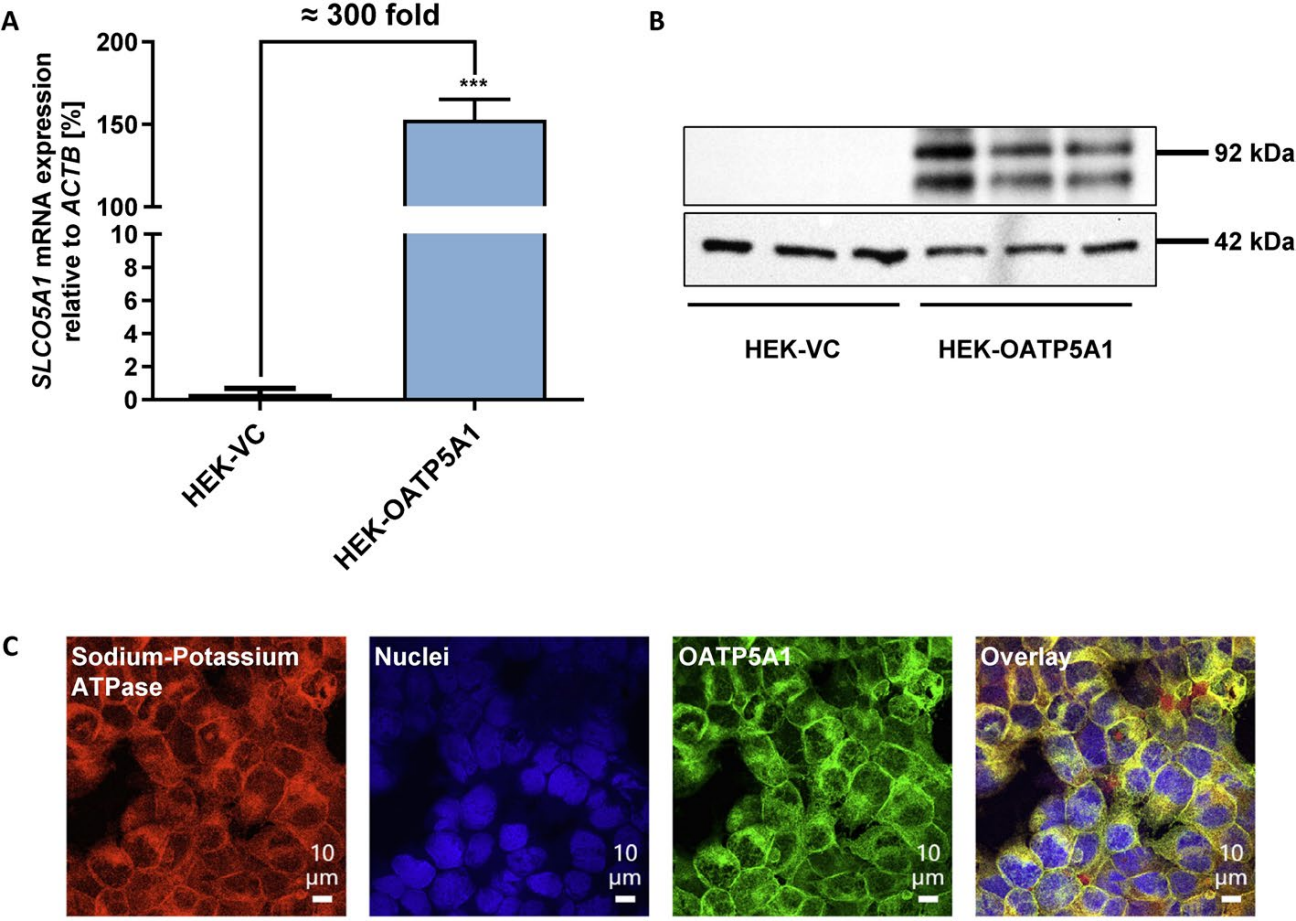
Characterisation of HEK293 cells stably overexpressing OATP5A1. **A**   *SLCO5A1* mRNA expression analysis demonstrated a highly significant *SLCO5A1* overexpression in the HEK-OATP5A1 cells compared to HEK-VC cells (149.6 ± 11.1% related to the expression of the housekeeping gene *ACTB* in the transfected cell line vs. an expression of 0.5 ± 0.2% in the control cell line). Data are displayed as mean ± SEM. ***p < 0.001 HEK-OATP5A1 versus HEK-VC. The experiment was performed with three independent biological replicates. **B** Immunoblot analysis of whole cell homogenates of HEK-VC cells and HEK-OATP5A1 cells with three biological replicates, respectively (cropped). OATP5A1 (92 kDa) was detected using the polyclonal antiserum SSS, β-actin (42 kDa) served as loading control. **C** Localisation of OATP5A1 in transfected HEK293 cells using confocal laser scanning microscopy. The red colour shows the staining of the membrane marker sodium–potassium ATPase indicating membrane localisation, the blue colour represents the nuclei stained with DAPI and the green colour marks OATP5A1. Membrane localisation of OATP5A1 was confirmed by the yellow colour in the overlay. This figure shows a representative image from three biological replicates

HEK293 cells stably overexpressing human OATP5A1 were characterised by qRT-PCR, immunoblot and immunofluorescence analysis. qRT-PCR demonstrated a significantly higher *SLCO5A1* mRNA expression in HEK-OATP5A1 cells compared with the expression in HEK-VC cells (149.6 ± 11.1% versus 0.5 ± 0.2% = ≈300-fold overexpression related to the expression of the housekeeping gene *ACTB*, *p* < 0.001; Fig. 1A). In line with the mRNA expression data, immunoblot analysis demonstrated OATP5A1 protein expression only in HEK-OATP5A1 cells (Fig. 1B; for raw image file see Additional file 1). Localisation of the OATP5A1 protein was analysed by immunofluorescence analysis showing a localisation of the recombinantly expressed protein in the plasma membrane of HEK-OATP5A1 cells (Fig. 1C). Furthermore, other transporters of the OATP family or known to transport amino acids or vitamins showed a low endogenous expression at comparable levels in both cells (Additional file 3).

### Identification of OATP5A1 candidate substrates by uptake assays

**Fig. 2:**
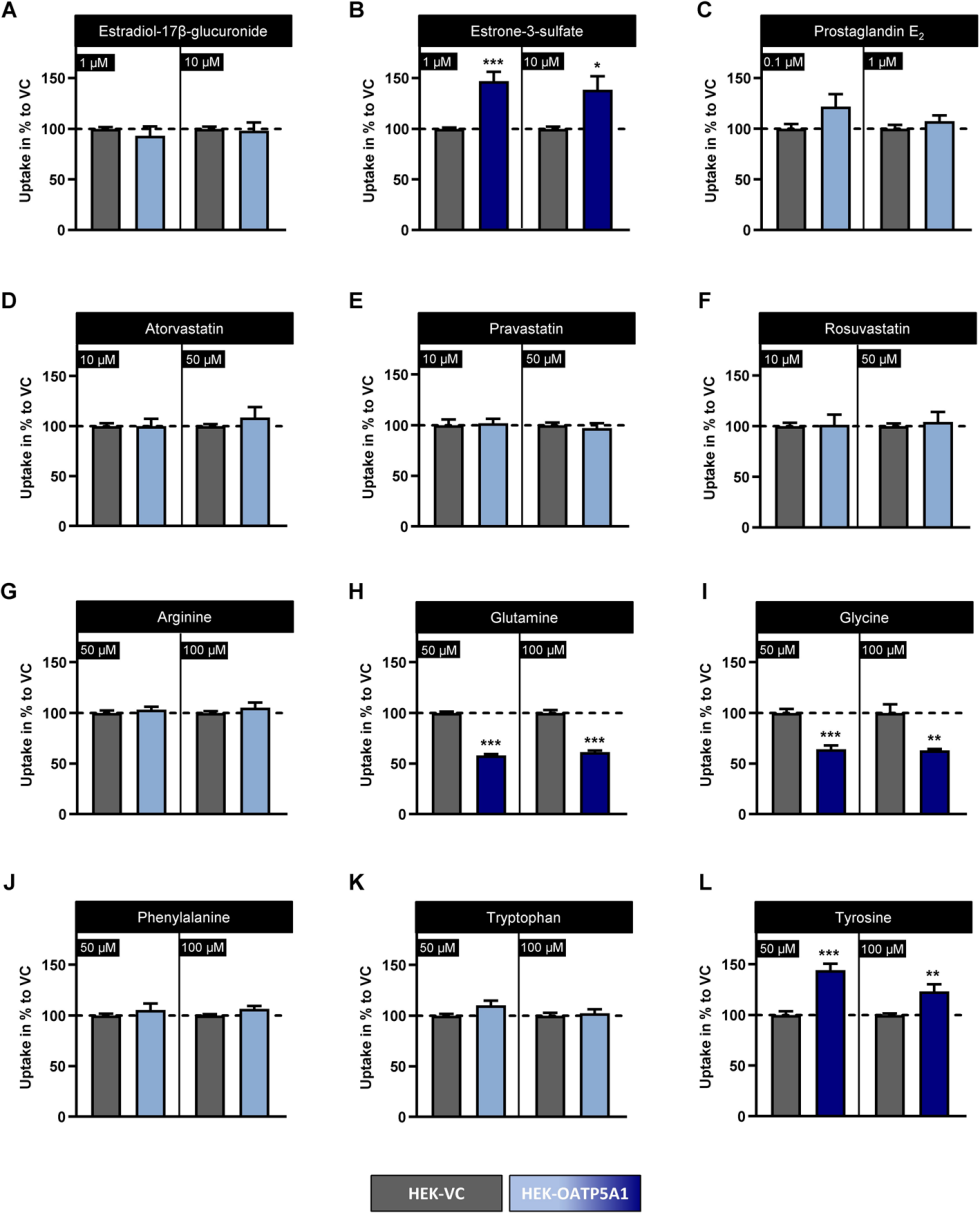
Transport studies using substrates of other OATP family members and amino acids. Uptake studies of radiolabelled substrates of OATP family members (**A**–**F**) and amino acids (**G**–**L**) using HEK-OATP5A1 cells (blue bars) and HEK-VC cells (grey bars). Intracellular accumulation was determined after 10 min of incubation for two concentrations. Substances with significantly different uptake into HEK-OATP5A1 cells compared with HEK-VC cells are indicated by dark blue bars. Data are shown as mean ± SEM of the uptake in HEK-VC. *p < 0.05; **p < 0.01; ***p < 0.001 versus HEK-VC. Experiments were performed with six independent biological replicates

Using the characterised HEK-OATP5A1 cells and the respective HEK-VC cells, we first investigated whether substrates of other OATPs are also substrates of human OATP5A1 (Fig. 2). We tested several statins (substrates, e.g., of OATP1B1 and OATP1B3), hormone metabolites (substrates, e.g., of OATP1A2 and OATP2B1) and amino acids (substrates, e.g., of OATP3A1) in established uptake assays. For most of the tested substances, no significant differences in the intracellular concentrations between HEK-OATP5A1 cells and HEK-VC cells were detected (Fig. 2). Significant OATP5A1-mediated uptake could be shown for estrone-3-sulfate (Fig. 2B) and the amino acid tyrosine (Fig. 2L). Interestingly, a highly significant lower intracellular concentration in HEK-OATP5A1 cells compared with HEK-VC cells was detected for the amino acids glutamine and glycine (Fig. 2H and I). Table 2 summarises the transport values of the four identified candidate substrates.

**Table 2:** Uptake of candidate substrates into HEK-OATP5A1 cells in comparison with HEK-VC cells

Substrates	Concentration [µM]	Uptake [% versus VC]
Estrone-3-sulfate	1	147.0 ± 9.2
	10	138.6 ± 13.3
Tyrosine	50	144.0 ± 7.4
	100	123.3 ± 6.9
Glutamine	50	58.0 ± 1.3
	100	61.3 ± 1.5
Glycine	50	64.2 ± 3.7
	100	63.0 ± 1.5

The uptake into HEK-OATP5A1 cells was normalised to the uptake in HEK-VC cells (100%). Each experiment was performed with six independent biological replicates after 10 min of incubation. Data are presented as mean ± SEM. For all substrates and concentrations, the difference between HEK-OATP5A1 and HEK-VC cells was statistically significant (*p* < 0.05)

### Identification of candidate substrates by untargeted metabolomics (LC–MS) analysis

**Fig. 3:**
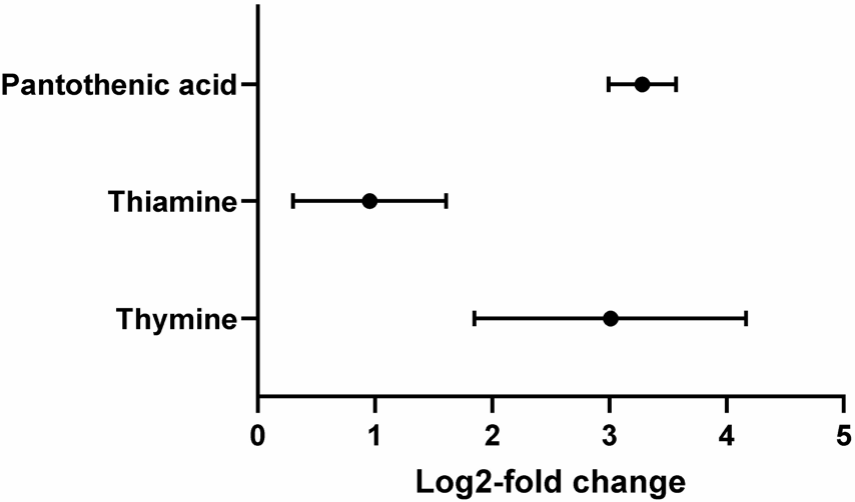
Untargeted metabolomics analysis of HEK-OATP5A1 and HEK-VC cells. Forest plot of log2-fold changes of pantothenic acid, thiamine and thymine identified being significantly more abundant in HEK-OATP5A1 cells compared with HEK-VC (log2-fold change > 1; 95% confidence intervals exclude log2-fold change = 0 indicating statistical significance). Cells were incubated with human plasma before analysis. Filled circles indicate the mean log2-fold change. Bars indicate the 95% confidence intervals of the mean log2-fold change. Experiments were performed with five independent biological replicates

In another approach to gain insights into the substrate spectrum of OATP5A1, we performed an untargeted metabolomics LC–MS analysis using both cell lines and human plasma as uptake matrix. This analysis showed significant differences in the intracellular concentrations of pantothenic acid, thiamine and thymine with all three substances being significantly more abundant in HEK-OATP5A1 compared with HEK-VC cells (Fig. 3).

### Characterisation of candidate OATP5A1 substrates

**Fig. 4:**
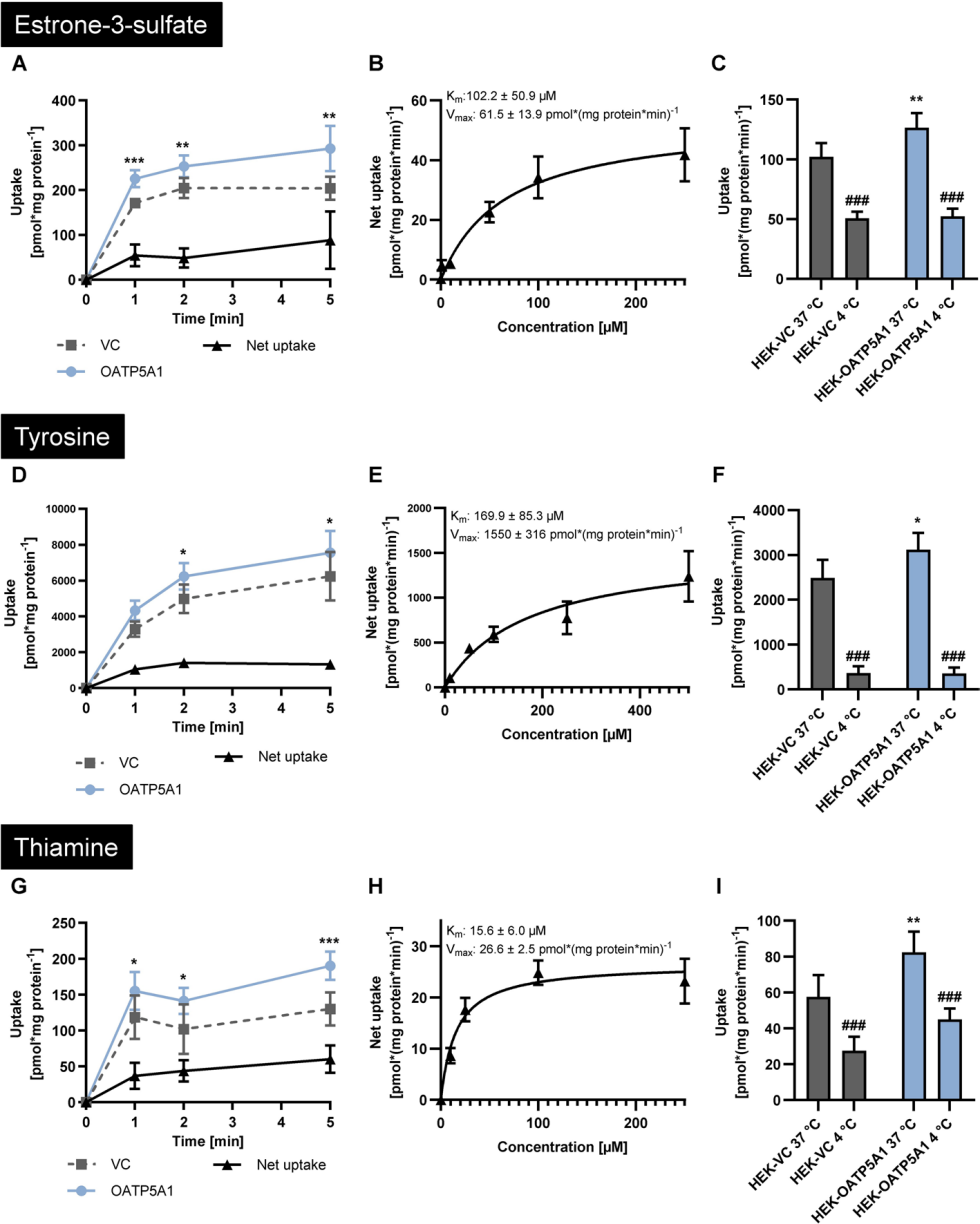
Uptake of externally administered estrone-3-sulfate, tyrosine and thiamine. Time-dependent uptake of estrone-3-sulfate (**A**), tyrosine (**D**) and thiamine (**G**) into HEK-OATP5A1 cells and HEK-VC cells was measured for 1, 2 and 5 min. The kinetic transport parameters (K_m_ values) and the maximal transport velocity (V_max_ values) were determined after 2 min of incubation (**B**, **E** and **H**). Influence of temperature on the uptake of estrone-3-sulfate (**C**), tyrosine (**F**) and thiamine (**I**) at 4 °C for 2 min compared with the uptake after incubation under equal conditions at 37 °C. Each time-dependent and temperature-dependent experiment was performed with substrate concentrations of 50 µM (estrone-3-sulfate) or 100 µM (tyrosine and thiamine). Data are shown as mean ± SEM. *p < 0.05; **p < 0.01; ***p < 0.001 HEK-OATP5A1 versus HEK-VC. ###*p* < 0.001 cell line with an incubation temperature of 4 °C versus the same cells with an incubation temperature of 37 °C, respectively. Experiments were performed with six independent biological replicates

Both approaches mentioned above thus led to the identification of seven potential OATP5A1 substrates. Using radiolabelled estrone-3-sulfate, radiolabelled tyrosine and unlabelled thiamine, we next investigated OATP5A1-mediated time-dependent (Fig. 4A, D and G) and concentration-dependent uptake, calculated kinetic transport constants (Fig. 4B, E and H) and performed inhibition studies using prototypic OATP substrates and transport inhibitors. Because of the high intracellular background of thiamine, cells were cultured in thiamine-free medium before uptake experiments. K_m_ values were 102.2 ± 50.9 µM; 169.9 ± 85.3 µM and 15.6 ± 6.0 µM for estrone-3-sulfate, tyrosine and thiamine, respectively. In addition, temperature-dependent experiments demonstrated that OATP5A1-mediated uptake of the three substances was significantly reduced at 4 °C (Fig. 4C, F and I). Furthermore, OATP5A1-mediated tyrosine uptake could be significantly inhibited by BSP, benzbromarone and to a lower extend by rifampicin (Additional file 2).

**Fig. 5:**
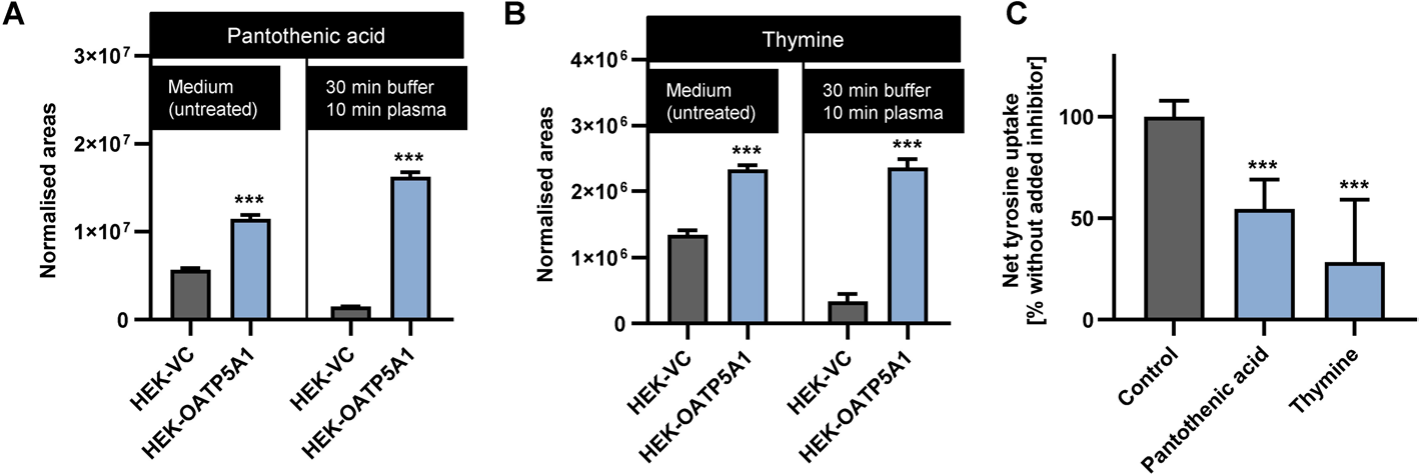
Intracellular concentrations of thymine and pantothenic acid without addition of exogenous substrates. Intracellular abundance of pantothenic acid (**A**) and thymine (**B**) in cells cultured in medium (analysed with LC–MS) and in cells after incubation for 30 min with uptake buffer followed by 10 min incubation with human plasma (analysed in untargeted metabolomics analysis) demonstrated significantly higher accumulation of the compounds in HEK-OATP5A1 cells. No additional exogenous pantothenic acid or thymine was added in these experiments. Inhibition of OATP5A1-mediated tyrosine uptake (100 µM) by 1 mM of pantothenic acid and thymine, respectively (**C**). Data are shown as mean ± SEM. ***p < 0.001 versus HEK-VC. LC–MS analysis and untargeted metabolomics were performed with five independent biological replicates, the inhibition experiments were performed with six independent biological replicates

The same experimental setup using labelled, externally added substances was applied for pantothenic acid and thymine. Both substances were exogenously added in two concentrations, respectively (10 µM and 50 µM for pantothenic acid, 1 µM and 10 µM for thymine). However, no significant uptake into HEK-OATP5A1 cells compared the HEK-VC cells could be detected even after starving the cells by culturing them in uptake buffer for 120 min. Because pantothenic acid and thymine are essential for cell survival, cell culture media without these compounds were not available. Therefore, we determined the amount of endogenously present, unlabelled pantothenic acid (Fig. 5A) and thymine (Fig. 5B) in untreated cells (cultured in medium) and in cells cultured for 30 min in uptake buffer and 10 min in human plasma (which were also used for untargeted metabolomics analysis). We could demonstrate that the endogenously present background amount of thymine and pantothenic acid was significantly higher in HEK-OATP5A1 cells in both untreated cells and after the incubation in uptake buffer and human plasma, compared with the amounts in HEK-VC cells (pantothenic acid: twofold after culturing in medium, 11-fold after incubation with uptake buffer and human plasma; thymine: twofold after culturing in medium, sevenfold after incubation with uptake buffer and human plasma, Fig. 5A and B). Moreover, inhibition experiments demonstrated that pantothenic acid and thymine significantly inhibited OATP5A1-mediated uptake of the OATP5A1 substrate tyrosine (Fig. 5C).

Taken together, these data indicate that pantothenic acid and thymine are also likely substrates of OATP5A1. Owing to the relatively high intracellular background levels of both compounds in HEK-OATP5A1 cells without exogenous addition of pantothenic acid or thymine (Fig. 5A and B), these high intracellular concentrations make it difficult to detect uptake of additional, externally administered substrates by facilitated diffusion. This likely explains that no OATP5A1-mediated uptake could be observed after exogenous addition of pantothenic acid or thymine.

### OATP5A1-mediated efflux of glutamine and glycine

**Fig. 6:**
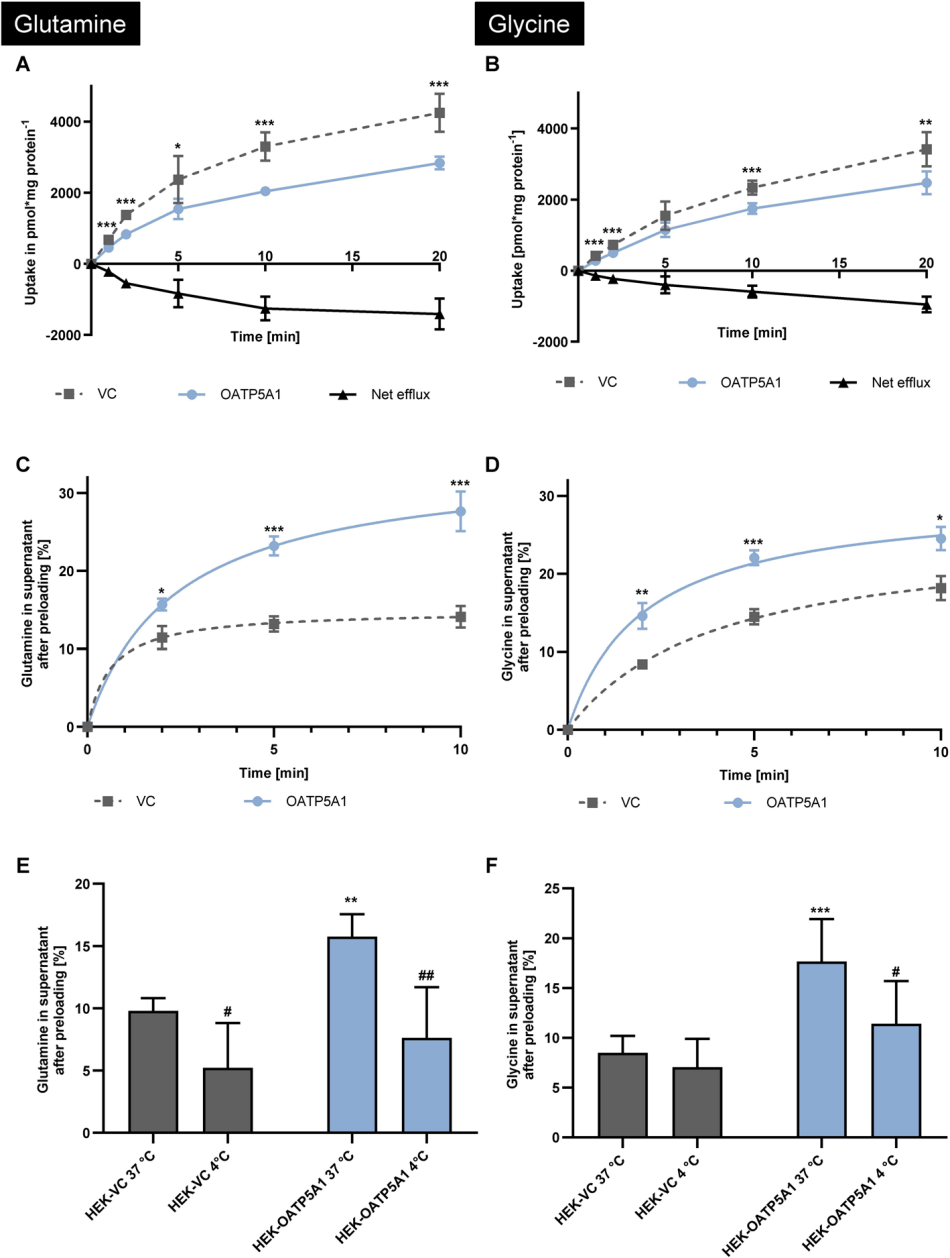
OATP5A1-mediated efflux of glutamine and glycine. Intracellular abundance of glutamine (**A**) and glycine (**B**) in HEK-OATP5A1 cells and HEK-VC cells after standardised uptake experiments with 50 µM for each amino acid, respectively. Net efflux demonstrated reduced amounts of glutamine and glycine in HEK-OATP5A1 cells. OATP5A1-mediated efflux of glutamine (**C**) and glycine (**D**) after preloading the cells for 60 min with 2 mM of each amino acid. Influence of the temperature on substrate transport of glutamine (**E**) and glycine (**F**) demonstrated reduced efflux at 4 °C after 2 min compared with 37 °C. Data are shown as mean ± SEM. *p < 0.05; **p ≤ 0.01; ***p < 0.001 HEK-OATP5A1 versus HEK-VC. ^#^*p* < 0.05; ^##^*p* < 0.01 cell line with an incubation temperature of 4 °C versus the same cells with an incubation temperature of 37 °C, respectively. Experiments were performed with six independent biological replicates

Because transport data for glutamine (Fig. 2H) and glycine (Fig. 2I) showed a reduced intracellular abundance in HEK-OATP5A1 versus HEK-VC, we hypothesised that OATP5A1 mediates efflux rather than uptake for these compounds. We investigated this using standardised uptake experiments and efflux experiments after preloading the cells (Fig. 6). The time-dependent uptake experiments for glutamine (Fig. 6A) and glycine (Fig. 6B) confirmed the initial uptake experiments (Fig. 2H and I) resulting in reduced intracellular concentrations in HEK-OATP5A1 versus HEK-VC cells. In line with these data, efflux experiments after preloading the cells (Fig. 6C and D) demonstrated a highly significant OATP5A1-mediated efflux which is significantly reduced in HEK-OATP5A1 cells by performing the same experiments at 4 °C (*p* < 0.05; Fig. 6E and F) confirming an OATP5A1-mediated efflux of glutamine and glycine.

## Discussion and conclusions

To assess the role of a transport protein in physiology or for pharmacokinetics, it is essential to determine its substrate spectrum. Unlike the well-characterised members of the ABC transporter superfamily, many SLC transporters are still classified as orphan transporters owing to a lack of data on their substrate spectrum and physiological function. To date, 70 different SLC families comprising more than 400 different transport proteins are known [3]. However, over 120 of these SLC transporters within the human genome do not have assigned substrates [31] and in silico studies suggest that there are more than another 120 still unknown SLC or SLC-like transporters [6]. One of these orphan transporters without a characterised substrate spectrum is the *SLC21*/*SLCO* family member OATP5A1 (*SLCO5A1*), which is the focus of this analysis.

The characterisation of HEK cells recombinantly overexpressing human OATP5A1 demonstrated an approximately 300-fold overexpression of *SLCO5A1* mRNA in HEK-OATP5A1 cells compared with the expression in HEK-VC control cells. The immunoblot analysis showed a band pattern comparable to already published data [16] with bands at 92 kDa representing the calculated core protein and additional bands at ~125 and 145 kDa representing partially and fully glycosylated protein.

Using these stably-transfected HEK293 cells, we investigated several candidate substrates and demonstrated that estrone-3-sulfate, glutamine, glycine, thiamine and tyrosine are substrates of this transporter. Furthermore, in the untargeted metabolomics analysis, pantothenic acid and thymine accumulated in HEK-OATP5A1 cells compared with HEK-VC cells after incubation for 30 min in uptake buffer and 10 min in human plasma without exogenous compound administration (11- and sevenfold accumulation ratios for pantothenic acid and thymine, respectively; *p*-value < 0.05). Both substances also inhibited OATP5A1-mediated tyrosine uptake, suggesting that they are substrates of OATP5A1 as well. The reason we could not investigate the transport for those substances using established methods with additional exogenous compound administration appears to be owing to the considerably higher intracellular background concentrations in the HEK-OATP5A1 cells compared with HEK-VC cells at the start of the uptake experiments (Fig. 5A and B). Culturing the cells in cell culture medium or incubating them in human plasma resulted in such high intracellular background concentrations of both substances that, consequently, it is likely that externally administered substrate does not lead to further increased uptake. No cell culture medium without pantothenic acid or thymine was commercially available, since these substances are essential for cell survival. For this reason, cells could not be cultured under standardised conditions over time without both substances.

The hormone conjugate estrone-3-sulfate has been investigated as substrate of OATP5A1 before. However, no significantly increased uptake was detected in these studies [16, 17]. The discrepancy to our findings may be owing to the differences in experimental conditions, including variations in the investigated concentrations of estrone-3-sulfate, as well as the utilisation of different cell systems, such as *X. laevis* oocytes instead of HEK293 cells. The K_m_ value for OATP5A1-mediated estrone-3-sulfate transport (102.2 µM) is higher than the K_m_ value for estrone-3-sulfate transport by other OATP family members (e.g., 0.46 µM for OATP1B1) [32]. This suggests that OATP5A1 is a low-affinity-high-capacity transporter of estrone-3-sulfate. Nevertheless, elevated expression levels of OATP5A1 may enhance the uptake of this hormone conjugate. Higher uptake of estrone-3-sulfate was detected in hormone-dependent breast cancer tissue compared with tissues from hormone-independent cancer owing to higher expression of OATPs [14, 33]. Estrone-3-sulfate can stimulate the proliferation of cancerous cells [34] because of its role as a source of oestrogen in hormone-dependent cancer cells. In this process, estrone-3-sulfate is metabolised by sulfatases into its active form estradiol, leading to increased tumour growth [33, 35, 36]. If OATP5A1-mediated transport is relevant under physiological conditions or in tumorous tissues after upregulation even though the K_m_ value is higher than expected, plasma concentrations should be investigated in further studies.

Thiamine (vitamin B_1_) uptake is mediated by the human thiamine transporters 1 (THTR1; *SLC19A2*) and 2 (THTR2; *SLC19A3*) and by the drug transporter OCT1 (*SLC22A1*) [37]. The K_m_ value for OATP5A1-mediated thiamine transport (15.6 µM) is between the high-affinity K_m_ values for THTR1 and THTR2 (2.5 µM and 27 nM, respectively) [38, 39] and OCT1, which has a relatively high K_m_ value of 780 µM [40] and also above the thiamine plasma concentrations of 40–120 nM [41]. However, since the concentrations of thiamine in the intestinal lumen can exceed concentrations of 100 µM [37] and because OATP5A1 is expressed throughout the human gastrointestinal tract [18, 19], this transport protein may contribute to the absorption of thiamine from food. Because thiamine is classified as an essential vitamin, the maintenance of health in humans depends on sufficient exogenous thiamine supply and protein-mediated uptake [42].

Since the OATP family member OATP3A1 has been shown to transport aromatic amino acids, we also investigated amino acids as candidate substrates for OATP5A1. Interestingly, only tyrosine was identified as an uptake substrate for both transporters with a slightly higher affinity for OATP5A1 (169.9 µM) than for OATP3A1 (220.8 µM) [9] suggesting that OATP5A1 plays a role in tyrosine homeostasis. Tyrosine uptake could be significantly inhibited by BSP (a substrate for several OATP family members), benzbromarone and, to a minor extent, by rifampicin.

In contrast to tyrosine, which is taken up by OATP5A1, the neutral amino acids glutamine and glycine were exported by this transporter. This was demonstrated by the initial uptake experiments (Fig. 2) and verified by preloading and efflux studies (Fig. 6). Because OATP5A1 is also expressed in the human brain, including neuronal cells and astrocytes [18, 19], this efflux could have important physiological functions. It has been shown that glutamine affects the homeostasis of the neurotransmitters glutamate and γ-aminobutyric acid (GABA) through the glutamate/GABA-glutamine cycle, a process that occurs between glutamatergic neurons and astrocytes [43]. During this process, astrocytes release glutamine into the extracellular space, from where it is taken up by GABAergic and glutamatergic neurons as a precursor for GABA and glutamate biosynthesis [44]. So far, glutamine transport in the brain has been shown to be mainly mediated by the sodium-coupled neutral amino acid transporters SNAT3 (*SLC38A3*), SNAT5 (*SLC38A5*), ASCT2 (*SLC1A5*) and LAT2 (*SLC7A8*) [45]. Interestingly, quantitative transport analysis demonstrated that only 60% of the necessary glutamine is transported by these proteins, leaving 40% of the responsible transport protein(s) unknown [46]. Therefore, OATP5A1 may be an additional transporter involved in glutamine efflux from astrocytes.

In addition to GABA, glycine is classified as a major inhibitory neurotransmitter in the human brain [47]. The synaptic uptake of glycine in the brain is mainly mediated by glycine transporter type 1 (*SLC6A9*) and 2 (*SLC6A5*) [48]. Disturbances in the homeostasis of glutamine and glycine in the brain can lead to pathological conditions as it was observed for Alzheimer’s disease [49], developmental delays, epilepsy [48, 50, 51], hyperekplexia, neuropathic pain, schizophrenia and stroke [48]. Interestingly, a genome-wide association study identified an single-nucleotide polymorphism (SNP) that colocalises with the *SLCO5A1* gene (encoding human OATP5A1) correlating with impulsivity in juvenile myoclonic epilepsy [24]. Therefore, these data suggest that OATP5A1 might have an important physiological function in maintaining the homoeostasis of neurotransmitters in the human brain. To gain more insights into this topic, further research using, e.g., knockout animal models or cell lines endogenously expressing OATP5A1 should be performed.

The limitations of the results presented here lie primarily in the concentrations of the substances investigated. As the focus of this study was to identify new substances as substrates for OATP5A1, the investigated concentrations do not necessarily represent the physiological plasma concentration in each case. Furthermore, time points chosen for kinetic measurements (2 min) may not be in the initial phase of OATP5A1-mediated transport. This should be addressed in further studies.

In summary, we deorphanised the OATP5A1 protein by identifying important endogenous substances as substrates for OATP5A1 for the first time. In addition to demonstrating that OATP5A1 mediates the uptake of the hormone metabolite estrone-3-sulfate, the vitamin thiamine and the essential amino acid tyrosine, we also verified OATP5A1-mediated efflux of glutamine and glycine, which are essential for brain function. Further research is required to elucidate the impact that influencing OATP5A1-mediated transport of these substrates could have on various neuropathological conditions and other diseases (e.g., cancer).

## Supplementary Information

Additional file 1. Immunoblot of HEK-VC cells and HEK-OATP5A1 cells detecting human OATP5A1. Immunoblot of three biological replicates of each cell line representing the presence of OATP5A1 (92 kDa) only in the transfected cells (HEK-OATP5A1) and β-actin (42 kDa) in both cell lines (HEK-OATP5A1 and HEK-VC) for loading control.

Additional file 2. Inhibition of OATP5A1-mediated tyrosine uptake by OATP-inhibitors. Inhibition of OATP5A1-mediated radiolabelled tyrosine uptake (100 µM) by 100 µM and 1 mM of bromosulfophthalein (BSP), benzbromarone, cyclosporine A, and rifampicin, respectively. The net tyrosine uptake into HEK-OATP5A1 after two minutes of incubation was significantly reduced for both concentrations of BSP and benzbromarone, as well as for 1 mM of rifampicin. Data are shown as mean ± SEM. *p ≤ 0.05; **p ≤ 0.01; ***p ≤ 0.001 HEK-OATP5A1 vs. HEK-VC. The experiments were performed with six independent biological replicates.

Additional file 3. qPCR expression analysis of 14 transport proteins in HEK-VC cells and HEK-OATP5A1 cells. qPCR analysis of two biological replicates of each cell line demonstrated similar expression of OATPs and other uptake transporters for amino acids and vitamins in the transfected cell line (HEK-OATP5A1) compared to the vector control cell line (HEK-VC). The expression was related to the expression of the housekeeping gene ACTB, which encodes β-actin.

## Data Availability

All data generated or analysed during this study are included in this published article (and its supplementary information files).

## Abbreviations

ABC: ATP-binding cassette
BSA: Bovine serum albumin
DAPI: 4′,6-Diamidin-2-phenylindol
GABA: γ-Aminobutyric acid
HEK293: Human embryonic kidney cells
HILIC: Hydrophilic interaction liquid chromatography
K_m_: Kinetic transport parameter (Michaelis–Menten constant)
LC: Liquid chromatography
MEM: Minimum essential medium
MS: Mass spectrometer
OAT: Organic anion transporter
OATP: Organic anion transporting polypeptide
OCT: Organic cation transporter
PBS: Phosphate buffered saline
PGE_2_: Prostaglandin E_2_
RP: Reversed phase liquid chromatography
(q)RT-PCR: (Quantitative) reverse transcription-polymerase chain reaction
SDS: Sodium dodecyl sulphate
SEM: Standard error of the mean
SLC: Solute carrier
SNP: Single nucleotide polymorphism
THTR: Human thiamine transporter
V_max_: Maximal transport velocity
