# Supplementary material for: Deorphanisation and functional characterisation of OATP5A1 as transport protein for amino acids and vitamins
Source: Cell Mol Biol Lett. 2026 Jun 2;31:75. doi: 10.1186/s11658-026-00943-7 (PMC13231527; doi:10.1186/s11658-026-00943-7)
Supplement: Supplementary file 1 — Additional file 1. Immunoblot of HEK-VC cells and HEK-OATP5A1 cells detecting human OATP5A1. Immunoblot of three biological replicates of each cell line representing the presence of OATP5A1 (92 kDa) only in the transfected cells (HEK-OATP5A1) and β-actin (42 kDa) in both cell lines (HEK-OATP5A1 and HEK-VC) for loading control. [file 11658_2026_943_MOESM1_ESM.pdf]

Supplementary Figure 1: Immunoblot of HEK-VC cells and HEK-OATP5A1 cells detecting human OATP5A1

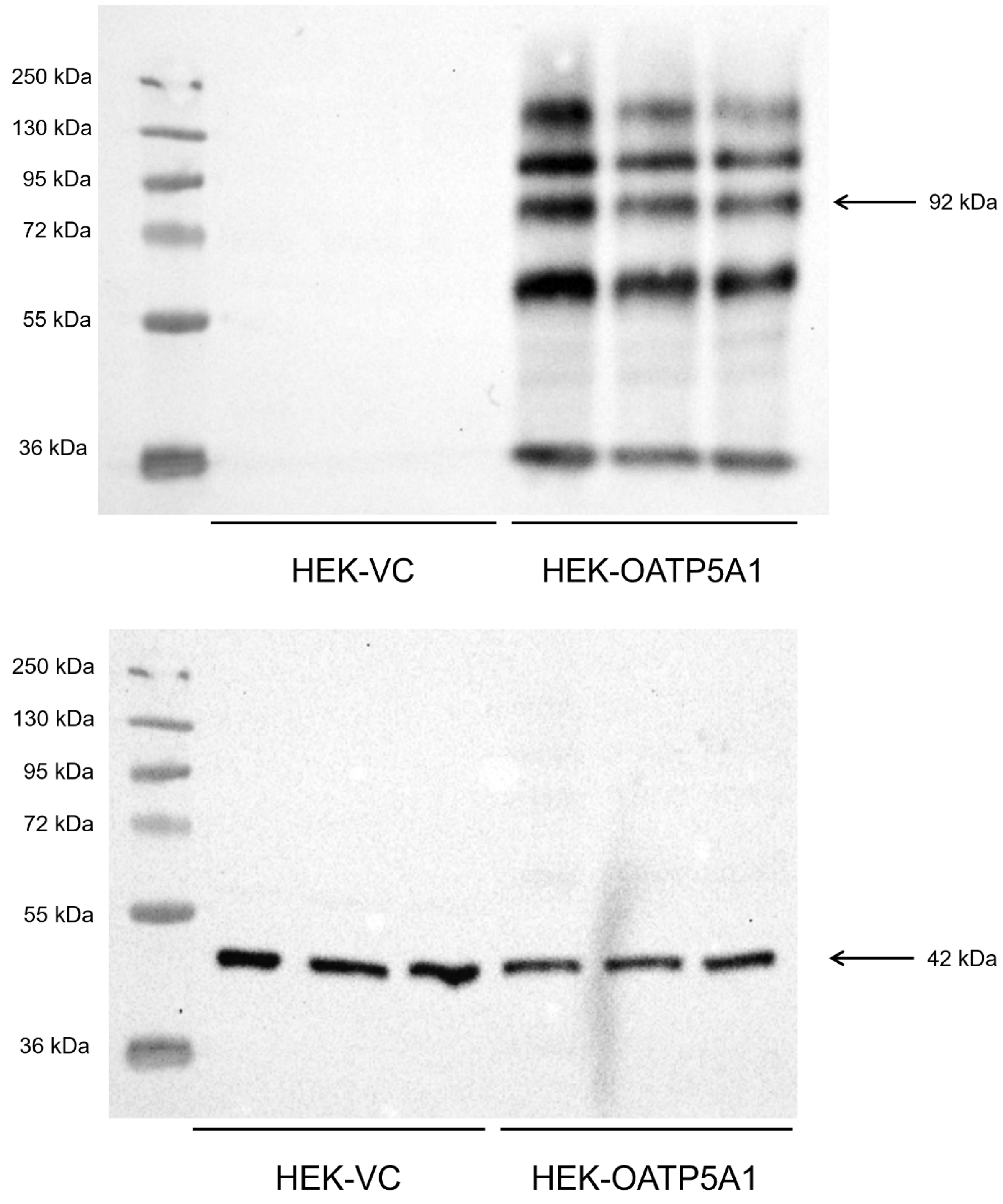

Immunoblot of three biological replicates of each cell line representing the presence of OATP5A1 (92 kDa) only in the transfected cells (HEK-OATP5A1) and β-actin (42 kDa) in both cell lines (HEK-OATP5A1 and HEK-VC) for loading control.
