# Supplementary material for: Deorphanisation and functional characterisation of OATP5A1 as transport protein for amino acids and vitamins
Source: Cell Mol Biol Lett. 2026 Jun 2;31:75. doi: 10.1186/s11658-026-00943-7 (PMC13231527; doi:10.1186/s11658-026-00943-7)
Supplement: Supplementary file 2 — Additional file 2. Inhibition of OATP5A1-mediated tyrosine uptake by OATP-inhibitors. Inhibition of OATP5A1-mediated radiolabelled tyrosine uptake (100 µM) by 100 µM and 1 mM of bromosulfophthalein (BSP), benzbromarone, cyclosporine A, and rifampicin, respectively. The net tyrosine uptake into HEK-OATP5A1 after two minutes of incubation was significantly reduced for both concentrations of BSP and benzbromarone, as well as for 1 mM of rifampicin. Data are shown as mean ± SEM. *p ≤ 0.05; **p ≤ 0.01; ***p ≤ 0.001 HEK-OATP5A1 vs. HEK-VC. The experiments were performed with six independent biological replicates. [file 11658_2026_943_MOESM2_ESM.pdf]

Supplementary Figure 2: Inhibition of OATP5A1-mediated tyrosine uptake by OATP-inhibitors

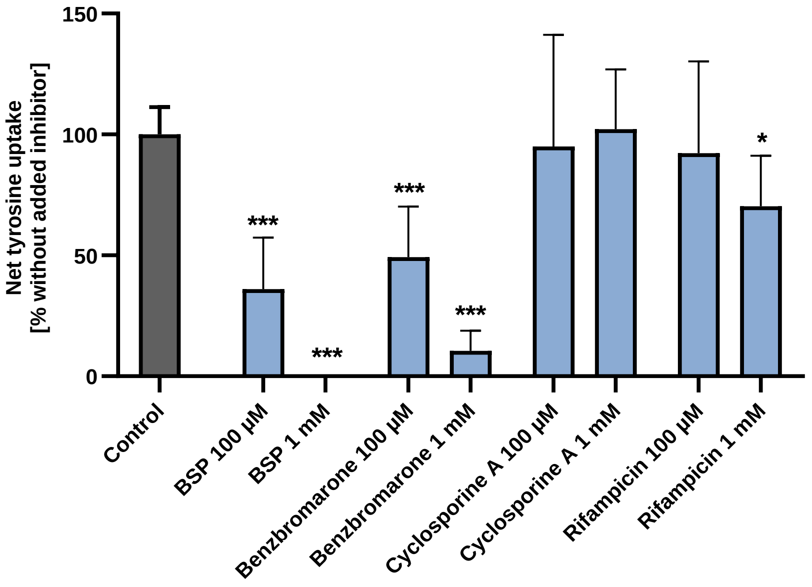

Inhibition of OATP5A1-mediated radiolabelled tyrosine uptake (100 µM) by 100 µM and 1 mM of bromosulfophthalein (BSP), benzbromarone, cyclosporine A, and rifampicin, respectively. The net tyrosine uptake into HEK-OATP5A1 after two minutes of incubation was significantly reduced for both concentrations of BSP and benzbromarone, as well as for 1 mM of rifampicin. Data are shown as mean ± SEM. $^{*}p \leq 0.05$; $^{**}p \leq 0.01$; $^{***}p \leq 0.001$ HEK-OATP5A1 vs. HEK-VC. The experiments were performed with six independent biological replicates.
