# Supplementary material for: Deorphanisation and functional characterisation of OATP5A1 as transport protein for amino acids and vitamins
Source: Cell Mol Biol Lett. 2026 Jun 2;31:75. doi: 10.1186/s11658-026-00943-7 (PMC13231527; doi:10.1186/s11658-026-00943-7)
Supplement: Supplementary file 3 — Additional file 3. qPCR expression analysis of 14 transport proteins in HEK-VC cells and HEK-OATP5A1 cells. qPCR analysis of two biological replicates of each cell line demonstrated similar expression of OATPs and other uptake transporters for amino acids and vitamins in the transfected cell line (HEK-OATP5A1) compared to the vector control cell line (HEK-VC). The expression was related to the expression of the housekeeping gene ACTB, which encodes β-actin. [file 11658_2026_943_MOESM3_ESM.pdf]

Supplementary Table 1: qPCR expression analysis of 14 transport proteins in HEK-VC cells and HEK-OATP5A1 cells

Transporter	Expression in HEK-VC [% to *ACTB*]	Expression in HEK-OATP5A1 [% to *ACTB*]	Forward primer sequence	Reverse primer sequence
**OATP1B1 (*SLCO1B1*)**	<0.1 %	<0.1 %	5'-tgc act tgg agg cac ctc ac-3'	5'-ctt cat cca tga cac ttc cat tt-3'
**OATP1B3 (*SLCO1B3*)**	<0.1 %	<0.1 %	5'-tca taa act ctt tgt tct ctg caa-3'	5'-gca tag act tat cca ttg gtc c-3'
**OATP2A1 (*SLCO2A1*)**	0.12 ± 0.016	<0.1 %	5'-cct cta cat gat ggt tct gcg-3'	5'-ggt cag atg agg cct gcc g-3'
**OATP2B1 (*SLCO2B1*)**	<0.1 %	<0.1 %	5'-tgc tca tcc taa gag gag tga a-3'	5'-ccc aag aca gct cac act cg-3'
**OATP3A1 (*SLCO3A1*)**	0.35 ± 0.084	0.16 ± 0.01	5'-tgc aac agc acg aat ctc ac-3'	5'-ccc tcc cca ggt tgt cta gg-3'
**OATP4A1 (*SLCO4A1*)**	0.41 ± 0.12	0.90 ± 0.04	5'-gag act gta gct gta tcc ctc-3'	5'-gcg gtg gtc aga cgc tgc t-3'
**OATP6A1 (*SLCO6A1*)**	<0.1 %	<0.1 %	5'-gat gca aag tgc tat aag tta cc-3'	5'-tcc agt tac aag tca gtt tct tc-3'
**SNAT3 (*SLC38A3*)**	<0.1 %	<0.1 %	5'-cct cat ctt cat ctt ccc tgc c-3'	5'-gtc tgg gca ggg ctg cta gg-3'
**SNAT5 (*SLC38A5*)**	<0.1 %	<0.1 %	5'-caa cca tcc ggg ata tct ttg ga-3'	5'-gag cgg ccc tga ccc ctc c-3'
**GLYT1 (*SLC6A9*)**	<0.1 %	<0.1 %	5'-cca tgt tcc ggc tct gcc gc-3'	5'-gtc tct gcg gtg gga gca cg-3'
**GLYT2 (*SLC6A5*)**	<0.1 %	<0.1 %	5'-ctg ttc cgt cat ctg gat ccc a-3'	5'-cag gat caa gtc tgg acc atc c-3'
**THTR1 (*SLC19A2*)**	0.80 ± 0.44	1.36 ± 0.02	5'-gca ctg cag acg ctg ctc act-3'	5'-gag ttc ttg cta taa gaa gaa gcc-3'
**THTR2 (*SLC19A3*)**	<0.1 %	<0.1 %	5'-tcc cag aag gat gta cag agc c-3'	5'-agt tat ggc aaa aca tat gcc acc-3'
**LAT1 (*SLC7A5*)**	<0.1 %	<0.1 %	5'-cct cgt gtt cac gtg tgt ga-3'	5'-aca gga cgg tcg tgg aga aga t-3'

qPCR analysis of two biological replicates of each cell line demonstrated similar expression of OATPs and other uptake transporters for amino acids and vitamins in the transfected cell line (HEK-OATP5A1) compared to the vector control cell line (HEK-VC). The expression was related to the expression of the housekeeping gene *ACTB*, which encodes β-actin.
